# Hybridoma-Derived Idiotype Vaccine for Lymphoma: Approval Must Wait

**DOI:** 10.3390/ph3030667

**Published:** 2010-03-15

**Authors:** Maurizio Bendandi

**Affiliations:** Center for Applied Medical Research and University Hospital, University of Navarra, Avda. Pio XII 36-55, 31008 Pamplona, Spain; Email: mbendandi@unav.es; Tel.: +34 948 194 700 x1004

**Keywords:** hybridoma, idiotype, immune response, lymphoma, randomized trials, recombinant, vaccine

## Abstract

Hybridoma-derived idiotype vaccines have been used for the experimental treatment of human lymphoma over the last twenty years, providing evidence of biological efficacy, clinical efficacy and clinical benefit. However, the product that has come closer to regulatory approval is unlikely to clear that hurdle due to the insufficiently robust data obtained in a recently closed clinical trial. This review aims at discussing the reasons for hybridoma-derived idiotype vaccines, more difficult to produce but also more successful than recombinant idiotype vaccines so far, are unlikely to gain regulatory approval. In particular, it is necessary to examine the many peculiar features of this therapeutic approach in a broader context, with special attention to concepts like customized active immunotherapy and randomization. Most published trials based on hybridoma-derived idiotype vaccines are being analyzed, together with the yet non-peer reviewed data from the only randomized study conducted so far with this product, and with the main trials on recombinant idiotype vaccines for thorough comparison. All in all, the sole randomized trial ever conducted on hybridoma-derived idiotype vaccines failed to achieve its primary clinical end point because of an insufficient accrual and because the statistical significance achieved was not as stringent as required for regulatory approval.

## Introduction

The estimated 66,000 new cases over this year make of non-Hodgkin’s lymphoma (NHL) the most common hematologic malignancy in the United States [1]. While significant epidemiological differences exist in different geographic regions, among B-cell NHL subtypes follicular lymphoma (FL) represents about 29% of cases, while mantle cell lymphoma (MCL) accounts for about 7% of them [2]. These two B-cell NHL subtypes are the sole in which hybridoma-derived idiotype vaccines have been tested so far, with the former having been the subject of the vast majority of such clinical trials [3]. However, at least in principle, any B-cell NHL subtype whose cells express on their surface either a complete clonal immunoglobulin or at least some of its idiotopes [4] could be treated with an idiotype vaccine with the aim of preventing disease relapse once a clinical response has been achieved through standard of care therapy [5].

While quite different from a clinical and biological standpoint, both FL and MCL are still considered incurable, and even though many more therapeutic tools are available for the former, indolent type of cancer [6], in both cases it is safe to state that a successful, innocuous [7], patient-specific and therefore customized vaccination strategy like that targeting the lymphoma-specific idiotype would definitely fulfill the criteria of responding to an unmet medical need. Indeed, upon demonstration of clinical efficacy and benefit, no other currently available treatment could compare with idiotypic vaccination in terms of safety profile [6]. 

### Overview of the market

Most cases of B-cell NHL remain incurable. However, the more indolent the subtype is, the more likely subsequent clinical remissions may be achieved through several therapeutic options. This is particularly true in the case of FL [8], the most classical field of application of idiotypic vaccination [3]. Over the last decade, the anti-CD20 monoclonal antibody rituximab has revolutionized the way oncologists treat FL, MCL and virtually any other CD20-positive B-cell NHL, to such an extent that the combination of rituximab and different chemotherapy regimens is now the standard of care for most if not all B-cell lymphoma [6]. In particular, with these combinations both higher response rates and longer response duration are systematically achieved compared to those obtained by chemotherapy alone [6]. Other novel agents, including new monoclonal antibodies targeting CD20 or other surface antigens [6], are also approaching the final stages of their development. However, neither rituximab nor any of them seem to have the potential to cure most if not all patients with indolent B-cell NHL. Many patients tend to develop resistance upon retreatment [9] and/or to suffer from the side effects. In this respect, even use of the generally safe rituximab is not entirely devoid of them, particularly when employed for an extensive period of time as a maintenance treatment. Progressive multifocal leukoencephalopathy is as rare as invariably and rapidly lethal, while the full implications of the long-term immune suppression associated with the use of rituximab has not yet been evaluated due to the relatively short follow-up of most trials on rituximab maintenance [6].

All in all, as mentioned above, we still need therapeutic tools that may be both curative and completely safe for at least a portion of patients with B-cell NHL. Idiotype vaccines are serious candidates to prove possessing both these features, and among them hybridoma-derived idiotype vaccines are those with the longest track record (Table 1), although none of them has yet either made it to the market or gained regulatory approval [3].

**Table 1:** Major studies on hybridoma-derived idiotype vaccine for lymphoma. Legend: Id: idiotype; KLH: keyhole limpet hemocyanin; FL: follicular lymphoma; MCL: mantle cell lymphoma.

Institution/Sponsor	Idiotype Production	Vaccine Formulation	Disease	Biological Efficacy	Clinical Efficacy	Clinical Benefit	Reference
Stanford University	Hybridoma	Id-KLH + SAF-1	FL	YES	N/A	N/A	[23]
NCI	Hybridoma	Id-KLH + GM-CSF	MCL	YES	N/A	N/A	[37]
NCI	Hybridoma	Id-KLH + GM-CSF	FL	YES	YES	N/A	[24]
Puerta de Hierro Hospital	Hybridoma	Id-KLH + GM-CSF	FL	YES	YES	N/A	[25]
University of Navarra	Hybridoma	Id-KLH + GM-CSF	FL	YES	N/A	YES	[26]
Biovest	Hybridoma/AutovaxId/BiovaxId	Id-KLH + GM-CSF	FL	pending	pending	YES	[31]

## Introduction to the Compound

Most idiotype vaccines, including those whose idiotype is rescued from hybridomas, currently consist of three ingredients [3]: an idiotype-containing immunoglobulin, an immunogenic carrier like keyhole limpet hemocyanin (KLH), and immunologic adjuvant like granulocyte-macrophage colony-stimulating factor (GM-CSF).

**Figure 1:**
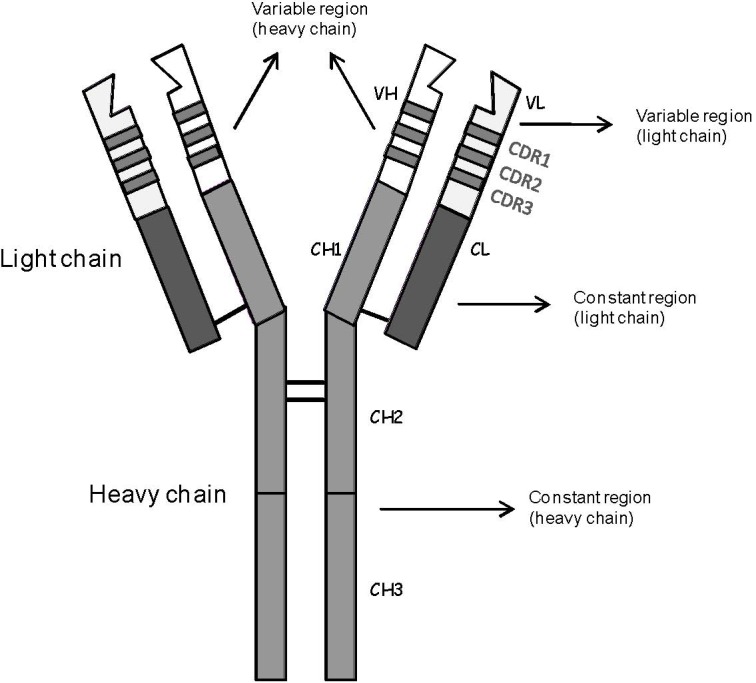
Schematic representation of the structure of a monomeric immunoglobulin. The collection of idiotopes termed idiotype is located within both heavy and light chain variable regions. VH: heavy chain variable region; VL: light chain variable region; CH: heavy chain constant region; CL: light chain constant region; CDR: complementarity-determining region.

The idiotype (Figure 1) is defined as the collection of idiotopes, that is the collection of all epitopes solely found on the hypervariable regions of an immunoglobulin [10,11]. As such, the idiotype defines the clonality of each and every immunoglobulin, including those expressed by B-cell NHL [3]. As a consequence, most B-cell NHL clones feature a patient- and tumor-specific idiotype, which can be used as cancer vaccine antigen and targeted in a customized fashion [12]. So far, the most important idiotype vaccine trials have been conducted using soluble protein, idiotype-containing full immunoglobulins [3]. 

KLH is a natural and powerful immunogenic carrier extracted from the mollusk Megathura Crenulata and used in humans now for decades without any type of clinical or biological concern [13].

GM-CSF is a hematopoietic growth factor that, besides stimulating myeloid cell maturation, is also known to attract dendritic cells at the site of an antigen injection (function not possessed by the more widely used alternative, that is G-CSF), and as such is commonly used as an immunologic adjuvant [14].

### Chemistry

While a few clinical studies have already been conducted using recombinant idiotype vaccines [3], the hybridoma-based rescue of patient- and tumor-specific idiotypes relies on an entirely different methodology [3], certainly more tedious and far less straightforward [15,16] even when partly streamlined and automated through the development of cell growth instruments [17]. However, at the time of this writing hybridoma-derived idiotype vaccines have shown biological efficacy, clinical efficacy and clinical benefit in lymphoma patients, while the recombinant alternatives have shown only biological efficacy [3].

As opposed to recombinant idiotypes, which are mounted on a shared, tumor- and potentially isotype-unrelated immunoglobulin heavy chain scaffold [3], hybridoma-methodology allows the reproduction in the lab of the original and unique, idiotype-containing tumor immunoglobulin [3]. In neither version of the tumor-specific idiotype, however, it is possible to reproduce the same idiotype glycosylation [18,19,20] pattern as it is present on the idiotype expressed on the surface of the lymphoma cell [3]. It is not fully known whether these types of difference may affect the immunogenicity of an idiotype vaccine and/or the relevance of a vaccine-induced immune response to the tumor antigen actually expressed on the tumor cell membrane. The limited data available in this respect seem to confirm that they do not [3], but further research is certainly warranted.

Once the tumor-specific idiotype has been reproduced, it is subjected to conjugation with KLH through the use of either glutharaldeide or maleimide [3]. So far, only the former has been employed for the development of clinical grade idiotype vaccines to be used in clinical trials. GM-CSF is administered separately subcutaneously over few days at the same site where the idiotype-KLH conjugate has been also administered subcutaneously on the first day of the vaccination cycle [3].

### Pharmacodynamics

Typically, idiotype vaccines are administered at a dose of 1 mg (0.5 mg of idiotype conjugated with 0.5 mg of KLH) on day 0 of each vaccination cycle, while GM-CSF is administered together with the vaccine at a dose which may vary from trial to trial [3]. The frequency and duration of these vaccination cycles may also vary from trial to trial [3], sometimes even constantly over several years [7].

As discussed elsewhere [7], an idiotype vaccine for lymphoma is not a drug in its common definition. It is rather a customized pharmacological agent meant to do per se little or nothing to both normal and tumor cells. The sole purpose of its combination of ingredients is that of eliciting an idiotype-specific immune response possibly capable of eliminating the tumor cells of one patient at a time. Irrespective of whether such an ambitious goal is or is not achieved by this type of customized active immunotherapy, the side effects are absent or minimal, self-limiting and typically local, although mild flu-like symptoms have also been reported in a minority of patients as due to GM-CSF [7].

### Pharmacokinetics and Metabolism

Similarly, the destine of any of the three proteins comprised in the vaccine formulation (idiotype, KLH and GM-CSF) is not expected to be different from that of any other vaccine protein administered subcutaneously, beginning with the uptake from professional antigen-presenting cells.

It has to be noted that both the self/non-self and the danger model [21] seem to fall short in explaining the basic way of functioning of idiotype vaccines. The former because it does not explain why it is still possible to allure the immune system into recognizing a self antigen, the idiotype, as if it were non-self by simply conjugating it with a bona fide non-self antigen like KLH. The latter because, assuming that repeated administrations of idiotype-KLH (where KLH is supposed to represent the danger signal) are responsible for the circumvention of the self/non-self issue, then this strategy should work in all patients endowed with a relatively normal immune function. However, this is not the case, as a non-negligible fraction of such vaccinated patients still fails to develop an idiotype-specific immune response in spite of normally reacting to KLH [10,11].

### Clinical efficacy and benefit

After showing biological efficacy [3] in several phase-I and -II studies over the last twenty years [22,23,24,25,26], hybridoma-derived idiotype vaccines have more recently shown also clinical efficacy [3] in a couple of phase-II trials [24,25] and even clinical benefit in a single phase-II trial [26]. In particular, it is now well established that most patients receiving a hybridoma-derived idiotype vaccine develop an idiotype-specific humoral and/or cellular immune response [22,23,24,25,26]. Moreover, these vaccine-induced, specific immune responses are often associated with the clearance *in vivo* [3] of tumor cells that had survived pre-vaccine chemotherapy [24,25]. Finally, a single study was designed to show that, contrary to the knowledge gathered over 50 years of clinical research in follicular lymphoma [27], immunization with a hybridoma-derived idiotype vaccine was capable of prolonging post-chemotherapy (without rituximab) second complete responses in FL patients well beyond its typical average duration (13 months), as well as beyond the duration of the post-chemotherapy (with/without rituximab) first complete response in each and every patient with a vaccine-induced, idiotype-specific immune response [26]. Indeed, 20/20 patients who developed such an immune response achieved this dual goal in a highly statistical fashion, while the 5/5 who did not develop such an immune response failed to achieve either result. Of course, this novel way to show clinical benefit of a customized type of active immunotherapy has raised some generic objections [28,29]. However, as discussed elsewhere [3,30], these criticisms cannot withstand a deeper and unbiased analysis of the actual clinical data.

The only phase-III trial ever conducted to test hybridoma-derived idiotype vaccines [31] has recently failed to achieve its main clinical endpoint for reasons that are completely unrelated to the actual vaccine effectiveness [3]. Appropriately designed to offer either the customized, soluble protein, hybridoma-derived idiotype vaccine (BiovaxId^TM^, Biovest International, Inc.) or a placebo control only to follicular lymphoma patients in first complete response [3], this study did not include a sufficiently effective and popular pre-vaccine chemotherapy regimen [3]. As a consequence, and as predicted [32], it ultimately failed to both enroll enough patients and to achieve the highly statistical significance required in this particular context for regulatory approval of this type of customized active immunotherapy [3]. In particular (Table 2), the trial enrolled 234 patients instead of the planned 629 (later revised to 563), randomized 177 (but only 117 ever made it to receive either the experimental or the control product) instead of 375, actually provided at least one dosis of the idiotype vaccine to 76 instead of 250, and had only 41 instead of 125 receiving the control product [31,32,33].

**Table 2:** Comparison between approval requirements and actual achievements of the phase-III randomized trial on BiovaxId^TM^. Legend: pts: patients; ITT: intent to treat; RFS: relapse-free survival.

Variable	Planned	Actual
Accrual	629 (later revised to 563) pts	234 pts
Randomization	375 pts	177 (later reduced to 117 on a modified ITT basis) pts
Vaccinated (5 doses)	250 pts	76 pts (at least 1 dose)
Control product	125 pts	41 pts
Required statistical significance (difference in RFS)	p < 0.01	p = 0.045

Notably, the vast majority of the 60 randomized patients who never had a chance to receive either treatment lost such an opportunity due to an early relapse [31]. Therefore, exactly 50% of all enrolled patients were unable to proceed with the crucial portion of the trial because pre-vaccine chemotherapy failed to induce or maintain a durable first complete response before vaccination could even start [3]. Since the data of this study have not yet been published in the peer-reviewed literature, it is impossible to ascertain how many patients actually completed the vaccination schedule and how many received fewer vaccinations than planned. In any case, the relapse-free survival of vaccinated patients was statistically significantly longer (p = 0.045) than that of patients who received the placebo control [31]. However, this statistical significance fell very short of the threshold of highly statistical significance (p, 0.01) previously established as necessary for regulatory approval, since no back-up trial had been planned [3].

All in all, the data of this trial further support the notion of clinical benefit of idiotypic vaccination in follicular lymphoma [26]. However, given the important pitfalls accumulated throughout the several years during which the study remained open, they are unlikely to provide ground for regulatory approval [3]. After all, had the trial succeeded in enrolling all planned patients, statistical significance of the difference in relapse-free survival could have improved, disappeared or remained unchanged [30].

### Safety and Tolerability

Several hundreds of patients have now being vaccinated with different types of recombinant and hybridoma-derived idiotype vaccine [3]. It is now clear that these products are very safe and tolerable, with negligible side effects even when administered periodically over several years [7].

### Regulatory affairs

Recently, all three randomized clinical trials (Table 3) conducted to confirm clinical benefit of idiotypic vaccination in follicular lymphoma, that is the study described above and two other using recombinant vaccines, have failed to achieve their main endpoints [31,34,35]. These predicted [32] and unfortunate outcomes could be explained on the basis of so many pitfalls intrinsic to each single study design and conduction that, nevertheless, it is impossible to firmly conclude whether any or all of these different vaccines [3] are or are not effective products [3,32,33].

**Table 3:** Main features of all randomized clinical trials on idiotypic vaccination. Legend: Legend: CR: complete response; PR: partial response; SD: stable disease; DFS: disease-free survival; TTP: time to progression; PFS: progression-free survival; ITT: intent to treat; n.s.: non-significant; CVP: cyclophosphamide, vincristine and prednisone; PACE: prednisone, doxorubicin, cyclophosphamide and etoposide.

Pre-Vaccine Therapy	Patient Status Before Vaccination	Accrual (planned / actual)	Endpoint	Results (required / obtained)	Reference
CVP (8 cycles)	First CR or PR	360 / 285	PFS	p < 0.01 / p = n.s.	34
Rituximab (4 doses)	First CR or PR or SD	342 / 345	TTP	p < 0.01 / p = n.s.	35
PACE (90% of pts) or Rituximab-CHOP (6 cycles)	First CR	375 / 117 (modified ITT)	DFS	p < 0.01 / p = 0.045	31

Even more important, the very same concept of randomization as a viable and meaningful tool to assess clinical benefit of idiotypic vaccination or any other customized active immunotherapy requires deeper and less biased questioning [3]. In facts, contrary to standard randomized trials where all patients within a same treatment group receive indeed the same therapy, in this case this statement holds true only for the control arm [8]. In the experimental arm, each and every patient receives a unique, different and differently immunogenic product, which is per se inactive against the tumor while supposed to stimulate a unique and different immune system that is in itself intrinsically prone to be more or less different at the time of each and every immunization [3]. In a sense, idiotypic vaccination, like any other customized active immunotherapy, challenges us with the ultimate “customized setting”, in which nearly all major biological variables - treatment (the idiotype), effector system (every time the vaccine is administered) and disease (particularly in the case of the clinically very heterogeneous follicular lymphoma) - are truly customized, and as such, arguably impossible to be subjected to the rigid constrains of randomization. In these extreme conditions, even studies theoretically well designed [31] may be ultimately doomed to fail simply because clearly unfeasible [32].

## Conclusions

After showing biological efficacy, clinical efficacy and clinical benefit [3], a hybridoma-derived idiotype vaccine is likely to fail to obtain regulatory approval for marketing due to the fact that the sole large-scale clinical trial that aimed at such a goal failed to achieve its main endpoint. With the substantial limitations of an insufficient patient accrual and of a limited statistical significance, this study nevertheless confirms the conclusions of a previous report of clinical benefit associated with idiotypic vaccination in follicular lymphoma [26]. 

The development of idiotype vaccines has temporarily come to an abrupt stalemate. Hybridoma methodology is unlikely to represent the basis for further testing of idiotypic vaccination in large scale clinical trials. Recombinant technology, in spite of the initial lack of success [34,35], may still provide ground for a renewed sense of hope for idiotype vaccines to be used in better designed and better conducted clinical trials [3]. In particular, new studies are being planned that employ recombinant idiotype proteins rapidly and effectively produced in plants [36]. Should these projects succeed, of course it would remain to be determined whether idiotypic vaccination may have a role in B-cell malignancies other than follicular lymphoma [37,38]. Most of these open questions might be answered over the next decade.

All in all, in the subset of patients with lymphoma in which it works, idiotypic vaccination is meant to represent a virtually innocuous maintenance treatment to be used after tumor shrinkage achieved by other pre-vaccine treatments: some to be preferred because non heavily immune suppressive, others not to because of their associated long-lasting B- or T-cell depletion [3,6]. In the maintenance setting, a successful idiotype vaccine would have to minimally compete with the far more toxic interferon and, above all, with the far more popular (although not devoid of relevant long-term toxicity) rituximab [3,6].

In most B-cell lymphoma, rituximab has shown best results when used in combination with standard chemotherapy in the first-line treatment, while its potential is clearly diminished upon re-usage at subsequent relapses [3,6]. Vice versa, idiotypic vaccination has shown its potential also in first relapse patients, provided that the subsequent pre-vaccine treatment was not accompanied by profound immune suppression. Therefore, should an idiotype vaccine make it to the market of indolent lymphoma, a potential long-term strategy might include both types of maintenance treatment at different time points. Alternatively, should the sequential use of rituximab (with or without chemotherapy) and idiotypic vaccination be considered within the same line of treatment, it might be important that, in the patients who do not experience an early relapse, substantial B-cell recovery be allowed before starting the latter procedure [3], although the evidence supporting this opinion is currently only indirect [35,37]. 

A major logistical difference between active (vaccine) and passive (rituximab and other monoclonal antibodies) immunotherapy of B-cell malignancies is that the former has to be provided on a customized basis and requires adequate tumor sample handling to assure that the individualized vaccine is properly produced, while the other is already available on an off-the-shelf basis.

Another aspect of idiotypic vaccination that will require further refinement is the way by which we assess and monitor vaccine-induced and idiotype-specific immune responses. Whether we refer to the arbitrary definitions of positive and negative humoral responses as measured by standard ELISA [30] or to the plethora of non-standardized methods used to document cellular responses [26], there is definitely room for conspicuous improvement.

Notwithstanding all these hurdles still to be overcome by idiotypic vaccination, the push to make them available to the patients remains unwavering, particularly considering that, as opposed to virtually any other cancer treatment, when effective it has basically no side effects; and when ineffective also [3].
